# Ceramide triggers p53-dependent apoptosis in genetically defined fibrosarcoma tumour cells

**DOI:** 10.1038/sj.bjc.6690411

**Published:** 1999-05

**Authors:** M Pruschy, H Resch, Y-Q Shi, N Aalame, C Glanzmann, S Bodis

**Affiliations:** Department of Radiation Oncology, Molecular Radiobiology Laboratory, Raemistrasse 100, Zurich, CH-8091, Switzerland

**Keywords:** p53, ceramide, sphingomyelin pathway, radiation therapy

## Abstract

p53 mutations are among the most common genetic alterations in human cancer and are frequently described in intrinsic or acquired radio- and chemotherapy resistance. Radiation-induced cell kill is not only mediated by DNA damage but also by the activation of signal transduction cascades generated at the plasma membrane like the sphingomyelin pathway. We used genetically defined wild-type p53 or p53-deficient mouse fibrosarcoma cells to investigate the p53-dependence of tumour response upon activation of the sphingomyelin pathway. Treatment of the tumour cells with neutral sphingomyelinase drastically reduced the amount of wild-type p53 fibrosarcoma cell proliferation over 72 h in a clear dose–response (0.2–1.0 U ml^−1^ nSMase). Sphingomyelinase had no effect on cell proliferation in tumour cells lacking p53. Similarly, cell proliferation was abolished by C2-ceramide (5–20 μM) only in wild-type p53 cells. FACS-analysis revealed that C2-ceramide induced massive p53-dependent apoptosis (40–50% after 12–24 h) and cell cycle analysis showed a transient G1 arrest in p53-deficient tumour cells 12–24 h after C2-ceramide exposure. These results suggest that ceramide-induced apoptosis in tumour cells can be dependent on the status of p53 and imply that p53 is also important for stress-induced apoptotic signal transduction cascades generated at the plasma membrane. © 1999 Cancer Research Campaign


*British Journal of Cancer* (1999) **80**(5/6), 693–698
© 1999 Cancer Research Campaign
Article no. bjoc.1998.0411

### Ceramide triggers p53-dependent apoptosis in genetically defined fibrosarcoma tumour cells

**M Pruschy, H Resch, Y-Q Shi, N Aalame, C Glanzmann and S Bodis**

Department of Radiation Oncology, Molecular Radiobiology Laboratory, University Hospital Zurich, Raemistrasse 100, CH-8091 Zurich, Switzerland

**Summary** p53 mutations are among the most common genetic alterations in human cancer and are frequently described in intrinsic or acquired radio- and chemotherapy resistance. Radiation-induced cell kill is not only mediated by DNA damage but also by the activation of signal transduction cascades generated at the plasma membrane like the sphingomyelin pathway. We used genetically defined wild-type p53 or p53-deficient mouse fibrosarcoma cells to investigate the p53-dependence of tumour response upon activation of the sphingomyelin pathway. Treatment of the tumour cells with neutral sphingomyelinase drastically reduced the amount of wild-type p53 fibrosarcoma cell proliferation over 72 h in a clear dose–response (0.2–1.0 U $ml^{-1}$ nSMase). Sphingomyelinase had no effect on cell proliferation in tumour cells lacking p53. Similarly, cell proliferation was abolished by C2-ceramide (5–20 $\mu$M) only in wild-type p53 cells. FACS-analysis revealed that C2-ceramide induced massive p53-dependent apoptosis (40–50% after 12–24 h) and cell cycle analysis showed a transient G1 arrest in p53-deficient tumour cells 12–24 h after C2-ceramide exposure. These results suggest that ceramide-induced apoptosis in tumour cells can be dependent on the status of p53 and imply that p53 is also important for stress-induced apoptotic signal transduction cascades generated at the plasma membrane.

**Keywords:** p53; ceramide; sphingomyelin pathway; radiation therapy

An increasing body of evidence indicates that spontaneous and treatment-induced apoptotic tumour cell death has wide implications for cancer therapy and as a prognostic factor (Wyllie, 1993; Hannun, 1997). This has led to reconsider the mechanisms of how tumour cells respond to cancer therapy (e.g. chemotherapy and ionizing radiation (IR) (Fisher, 1994). Induction of DNA double-strand breaks was considered as the major mechanism of IR-induced cell death. However, more recent studies focus on multiple IR-induced signal transduction cascades generated at the plasma membrane, that trigger cells to undergo apoptosis (Suzumiel, 1994; Maity et al, 1997).

The p53 tumour suppressor gene is a key element in the nucleus to regulate normal cell cycle activity and is often cited to be the ‘guardian of the genome’. p53 is one of the most frequently mutated genes in advanced human cancer and, if mutated, is often an inverse prognostic factor for outcome (Harris, 1996; Maity et al, 1997). p53 is activated upon DNA damage and, in response to other cytotoxic agents and can enhance the cytotoxic effect of IR through apoptosis induction in many human malignancies (Ruley, 1996).

The sphingomyelin pathway is an important apoptotic cell death pathway that is generated at the cell membrane by various triggers, e.g. IR, tumour necrosis factor (TNF)-$\alpha$ and Fas/Apo-1 (Obeid et al, 1993; Haimovitz-Friedman et al, 1994; Herr et al, 1997). This pathway is initiated by hydrolysis of sphingomyelin by specific phospholipases (sphingomyelinases) to generate the intracellular apoptotic second messenger ceramide. Activation of the sphingomyelin pathway and subsequent generation of ceramide by IR was shown to be independent of DNA damage (Haimovitz-Friedman et al, 1994). The second messenger ceramide has been shown to activate the stress-induced apoptotic JNK/SAPK cascade in some cell systems and is also involved in the coordination of cytokine-induced apoptotic pathways (Chinnaiyan et al, 1996; Verheij et al, 1996; Haimovitz-Friedman et al, 1997). Furthermore, ceramide has been shown to induce cytochrome C release from mitochondria in acute myeloid leukaemia cells to activate caspase 3-like activity (Amarante-Mendes et al, 1998).

In this study we investigated if the p53 status of tumour cells can modulate the apoptotic response upon activation of the sphingomyelin pathway. For this purpose we used a cell-permeable ceramide derivative or neutral sphingomyelinase (nSMase) in a genetically defined tumour system with a strict p53-dependent response to IR and various chemotherapeutic agents (Jacks et al, 1994; Lowe et al, 1994*a*).

*Received 26 August 1998*
*Revised 10 November 1998*
*Accepted 11 November 1998*

*Correspondence to:* S Bodis

### MATERIALS AND METHODS

### Cell culture and irradiation

Cell lines of p53+/+ and p53−/− mouse embryo fibroblasts (MEF) were derived from 13.5-day-old embryos and transformed by co-expression of E1A and T24 H-*ras* (referred to in the manuscript as *ras*) (Lowe et al, 1993*b*). These oncogenically transformed fibrosarcoma cells were used at low passage numbers and cultured at 5% carbon dioxide atmosphere in Dulbecco’s modified Eagle’s medium (DMEM) containing 10% fetal calf serum (FCS) and 10% bovine calf serum (BCS) (HyClone Laboratories) supplemented with penicillin and streptomycin. The characteristic p53-dependent response to ionizing radiation and apoptosis induction of these tumour cells were verified monthly. Irradiation of the cells was carried out at room temperature in tissue culture dishes (100 × 100 mm) with a 6 MV linear accelerator at a dose rate of

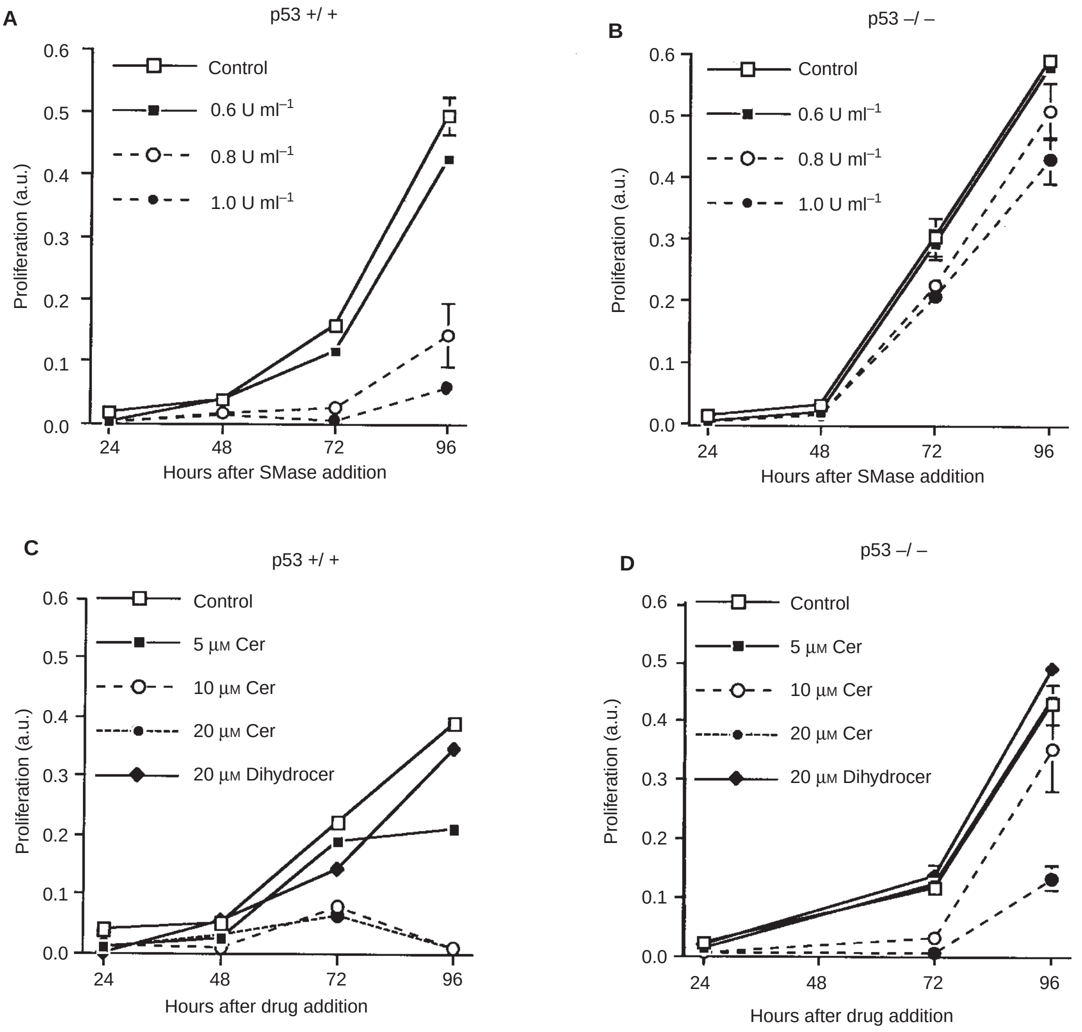

**Figure 1** Proliferation of fibrosarcoma cells after sphingomyelin pathway activation. Wild type p53 (**A**, **C**) and p53-deficient (**B**, **D**) fibrosarcoma cells were treated with increasing concentrations of nSMAse (**A**, **B**), C2-ceramide (Cer) or C2-dihydroceramide (Dihydrocer) (**C**, **D**) and proliferation was detected at different time points after drug addition with the alamarBlue assay. At least two independent experiments were performed in triplicates. Absence of error bars is due to minimal standard deviation

2 Gy per min. Dosimetry was controlled with a Vigilant-dosimeter. A 9 mm bolus on the tissue culture dishes was used for a build-up effect.

### Cell proliferation and clonogenic assay

The cell permeable C2-ceramide (*N*-acetylsphingosine) and the metabolically inactive C2-dihydroceramide (*N*-acetyldihydrosphingosine) were purchased from BIOMOL (Plymouth Meeting, PA, USA), the enzyme neutral sphingomyelinase derived from *Staphylococcus aureus* from Sigma (St Louis, MO, USA). Dissolved C2-ceramide (in dimethyl sulphoxide) and C2-dihydroceramide (in EtOH) was diluted in DMEM to the final concentration. nSMase was diluted in serum-free medium. For the experiments, cells were washed in serum-free medium and incubated with the drugs for 6 h in DMEM containing 0.5% FCS. After 6 h, serum was added to the final concentration of 20%. Tumour cell proliferation was assessed by the colourimetric alamarBlue assay that is based on detection of metabolite activity according to the protocol of the manufacturer (Biosource International, Camarillo, CA, USA). Absorption was measured at 570 and 600 nm using a Dynatech MR5000 spectrophotometer. To determine clonogenic survival, the number of singular cells plated was adjusted to obtain about 100 colonies per dish with a given treatment. After exposure to the different drugs, cells were maintained at 37°C in a humidified atmosphere containing 5% carbon dioxide. Cells were then allowed to grow for 8–10 days before fixation in methanol/acetic acid (75%/25%) and staining with crystal violet. Only colonies with more than 50 cells per colony were counted. The plating efficiency (PE) of untreated cells was determined and calculated by PE (%) = (scored colonies/number of plated cells) × 100. The surviving fraction (SF) with a given treatment was determined by SF = (scored colonies) / (number of plated cells × PE/100).

### Quantification of apoptosis and cell cycle analysis

Cells were prepared for apoptosis and cell cycle analysis using flow cytometry (Darzynkiewic, 1995). Briefly, adherent and floating cells were collected at different time points, fixed with

© Cancer Research Campaign 1999

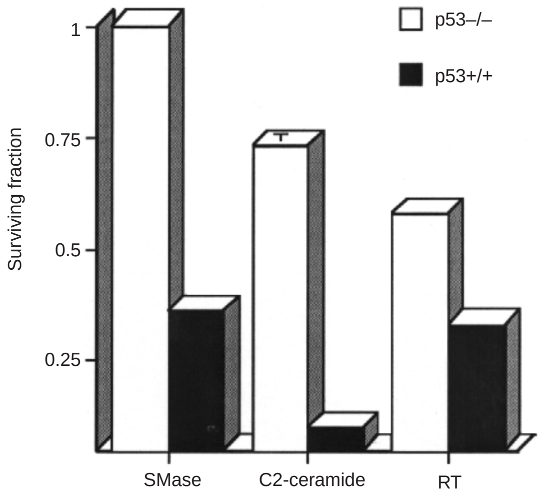

**Figure 2** Clonogenic survival of fibrosarcoma cells after sphingomyelin pathway activation and irradiation. Singular seeded p53+/+ and p53–/– cells were treated with nSMAse (1 U ml$^{-1}$), C2-ceramide (20 μM) or IR (5 Gy) and allowed to grow for 8–10 days. All experiments were performed in triplicates. Absence of error bars is due to minimal standard deviation

70% ethanol overnight at −20°C, washed with HBSS and resuspended in 1 ml of HBSS containing 50 u DNAase-free RNAase A per ml and propidium iodine (20 μg ml$^{-1}$). FACS analysis was performed on a FACScan (Becton Dickinson) and data of the different cell populations were analysed using Cell Quest software (Becton Dickinson). At least three independent experiments were performed for each set of data. For cell cycle quantification, the amount of apoptotic cells were subtracted from the total cell population and the amount of surviving cells are shown as 100%.

### RESULTS

### Inhibition of cell proliferation after sphingomyelin pathway activation is p53-dependent

Cell proliferation after induction of the sphingomyelin pathway was determined in transformed fibrosarcoma cells. This tumour cell system consists of E1A/*ras* transformed mouse embryo fibroblasts from either wild-type (+/+) or p53-knockout (–/–) mice and was previously described for its strict p53-dependent response to treatment with IR and different cytotoxic drugs in vitro and in vivo using nude mice as tumour carriers (Lowe et al, 1994*a*). Activation of the sphingomyelin pathway was achieved either by treatment of the cells with bacterial nSMase and subsequent generation of the apoptotic second messenger ceramide, or by the addition of membrane-permeable C2-ceramide.

Increasing doses of nSMase drastically reduced proliferation of wild-type p53 fibrosarcoma cells as assessed with the alamarBlue assay, a proliferation assay comparable to tetrazolium salt based (MTT-assay) quantification of cell metabolism. Proliferation was reduced in a clear dose–response and no metabolic activity was detected for at least 72 h after treatment with 0.8 U ml$^{-1}$ of SMase (Figure 1A). In the p53-deficient fibrosarcoma cells, cell proliferation was maximally reduced by 27%, even after treatment with high doses of SMase (Figure 1B).

In comparison to nSMase concentrations used in leukaemic cell lines for sphingomyelin pathway activation and apoptosis induction, tenfold higher concentration of nSMase (0.6 and 0.8 U ml$^{-1}$ of nSMase) was required to reduce the proliferation of the wild-type p53 fibrosarcoma cells. This could be due to a different lipid composition of this cell type or a restricted accessibility of nSMase to the cell membrane of adherent cells. Higher doses of nSMase were also required for sphingomyelinase-induced apoptosis in adherent bovine aortic endothelial cells (Haimovitz-Friedman et al, 1994).

To bypass differences in ceramide generation by nSMase due to possible variations of the cellular lipid composition, subsequent studies on cell proliferation were also performed with the synthetic and cell-permeable ceramide analogue *N*-acetyl-sphingosine (C2-ceramide). C2-ceramide (5 μM) reduced the amount of proliferation in the wild-type p53 tumour cells up to 50% and at higher concentrations of C2-ceramide (10 and 20 μM respectively), no proliferative activity could be detected for at least 96 h. These concentrations of C2-ceramide are in the range as previously used for other cell lines (Figure 1C) (Jarvis et al, 1996). A reduction of p53–/– tumour cell proliferation was only observed at the highest C2-ceramide concentration of 20 μM used for these experiments. Lower doses of C2-ceramide (5 and 10 μM) revealed no growth inhibition in this relatively radio- and chemoresistant cell line (Figure 1D). The metabolically inactive dihydro-C2-ceramide analogue had no effect on cell proliferation in both cell lines. Thus, decreased tumour cell proliferation after activation of the sphingomyelin pathway either by the nSMase or by cell-permeable C2-ceramide was dependent on the p53 status of the cell.

Next, we determined the effect of sphingomyelin pathway activation on the clonogenic survival of the wild-type p53 and p53-deficient tumour cells. Singular seeded wild-type p53 and p53-deficient fibrosarcoma cells were allowed to grow for 8–10 days in presence of nSMase (1 U ml$^{-1}$) or C2-ceramide (20 μM), at concentrations which revealed a distinct p53-dependent difference in proliferation as described above. Tumour cell colony formation of the wild-type p53 and p53-deficient cells after treatment with nSMase or C2-ceramide was compared to colony formation after irradiation (Figure 2). Whereas nSMase treatment of the p53–/– cells had almost no effect on the reproductive integrity, a reduction in colony formation of 25% and 40%, respectively, was observed after treatment with 20 μM C2-ceramide and 5 Gy IR. In the p53+/+ tumour cells, colony formation after nSMase and irradiation was reduced to 65% and C2-ceramide treatment reduced clonogenic survival up to 90% (mean values of several independent experiments, see legend to Figure 2). Thus, similar to the response to irradiation, sphingomyelin pathway activation strongly reduced clonogenic survival of wild-type p53 tumour cells but not of p53-deficient cells. Interestingly, the difference in cell survival between the two cell lines was even more distinct after sphingomyelin pathway activation than after treatment with 5 Gy IR.

We tested whether decreased proliferation and reduced clonogenic survival in the wild-type p53 tumour cells after ceramide exposure was due to p53-dependent induction of apoptosis. Characteristic morphological changes suggestive for apoptosis, such as chromatin condensation and nuclear fragmentation, were detected in the wild-type p53 cells after IR or C2-ceramide treatment and were only incidentally found in p53-deficient cells (data not shown). The amount of apoptosis in the different cell lines was quantified by flow cytometry. The p53+/+ and p53–/– fibrosarcoma cells were treated with C2-ceramide (20 μM) and analysed for apoptosis 12 and 24 h after drug addition. In the p53-deficient cell population, increase of ceramide-induced apoptosis was less than 5% of the total cell population at different time points (Figure

© Cancer Research Campaign 1999

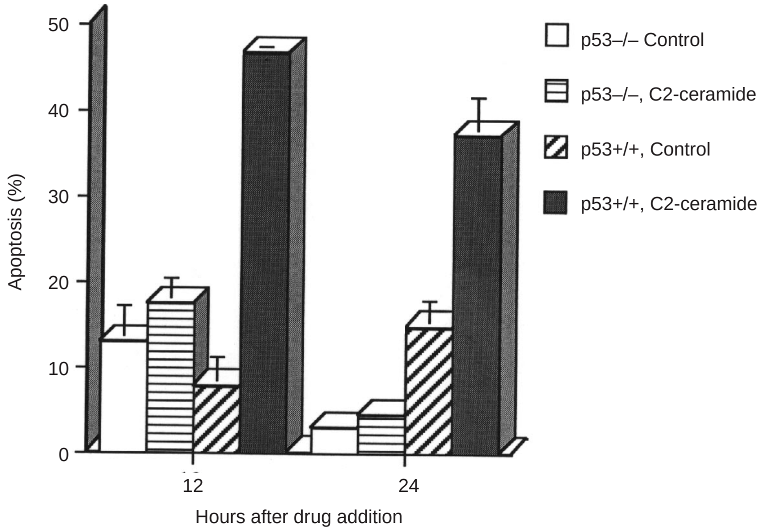

**Figure 3** C2-ceramide-induced apoptosis in fibrosarcoma cells as determined by flow cytometry. Tumour cells were incubated with C2-ceramide (20 μM) and the amount of apoptosis was determined 12 and 24 h, respectively, after drug addition. At least two independent experiments were performed

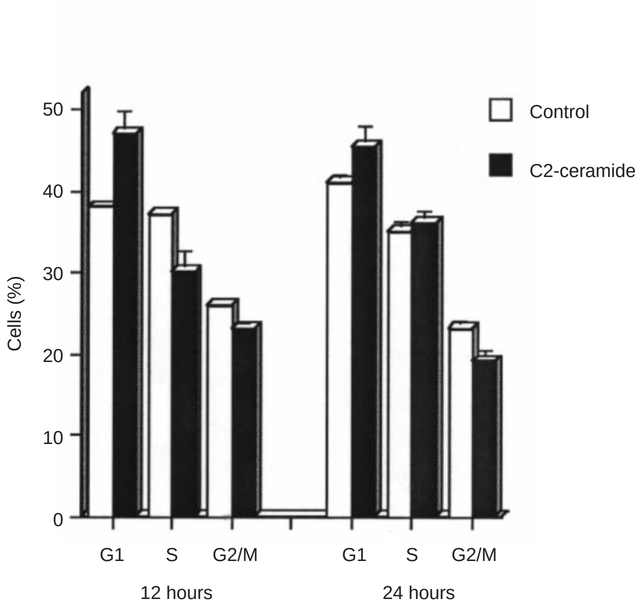

**Figure 4** Partial G1 arrest of p53–/– fibrosarcoma cells after C2-ceramide addition. p53–/– tumour cells were treated with C2-ceramide (20 μM) and cell cycle distribution was determined 12 and 24 h after drug addition. At least three independent experiments were performed and absence of error bars is due to minimal standard deviation

3). However, treatment of the p53+/+ cells with C2-ceramide increased the amount of apoptotic cells up to 45% of the whole cell population. Beyond 24 h, the amount of apoptotic cells declined (data not shown).

In addition to the induction of apoptosis, treatment of cells with ceramide can also lead to growth arrest. We examined the p53-deficient, but otherwise isogenic, E1A/*ras* transformed tumour cells for ceramide-induced cell cycle alterations. Treatment with C2-ceramide induced a transient accumulation of cells in G1 phase. The maximal accumulation of cells in the G1 phase was observed 12 h after C2-ceramide exposure (mean increase 37% (±0.6%) to 47% (±2.9%) and declined thereafter. In accordance with the increasing cell distribution into G1, a decreasing fraction of cells in S and G2/M phase was identified (Figure 4). Interestingly, we could not reveal any cell cycle alterations upon C2-ceramide exposure in the surviving wild-type p53 cell population that did not undergo apoptosis (data not shown).

### DISCUSSION

Activation of the sphingomyelin pathway specifically reduced proliferation and clonogenicity in the chemo- and radiation therapy-sensitive, wild-type p53 tumour cells due to the induction of apoptosis. These results imply that p53 is involved in the cellular response to ceramide, a specific inducer of apoptosis, that is independent of DNA damage.

The sphingomyelin–ceramide pathway can be activated by treatment of cells with TNF-α, low density lipoproteins and hydrogen peroxide. A partial p53-dependent effect on cell survival has been observed in differentiated macrophages treated with these agents (Kinscherf et al, 1998). Preincubation with p53-antisense oligonucleotides reduced oxidative stress-dependent up-regulation of p53 and decreased the amount of apoptosis by about 20%. In our cell system, we demonstrate that genetically defined tumour cells, differing only in their p53 status, have a different response to direct ceramide treatment. Ceramide strongly induced apoptosis in the wild-type p53, but not in the isogenic, p53-deficient, tumour cells.

Both p53+/+ and p53–/– mouse embryo fibroblasts are transformed by co-expression of E1A and *ras*. We can not exclude that transformation of these cells might lead to subsequent genetic alterations. But such changes could also reflect the processes in carcinogenesis in wild-type p53 and p53-mutated tumours leading to different phenotypes still primarily dependent on the p53 status for their treatment response.

Previously, Lowe et al (1994*b*) demonstrated that the p53 level in the wild-type p53 tumour cells used for these studies, is

 © Cancer Research Campaign 1999

up-regulated upon E1A/*ras* transformation. Serum depletion of the cells did not further increase the p53 level, but induced apoptosis in the wild-type p53 tumour cells (Lowe et al, 1994*b*). Growth factor withdrawal can result in significant sphingomyelin hydrolysis and progressive elevation of endogenous levels of ceramide (Hannun and Linardic, 1993; Jayadev et al, 1995). These findings suggest that ceramide might be the pivotal mediator for the observed p53-dependent apoptosis induction upon growth factor deprivation in this tumour cell system.

Stress factors may induce apoptosis by up-regulating pro-apoptotic or down-regulating anti-apoptotic processes. p53 is a direct transcriptional activator of the pro-apoptotic bax gene (Miyashita and Reed, 1995). In addition, growth factor deprivation and elevated ceramide levels result in the down-regulation of Akt kinase. Active Akt blocks BAD-mediated cell death via phosphorylation of BAD and, presumably, subsequent reduction of Bax-homodimer formation (Datta et al, 1997; Zhou et al, 1998; Zundel and Giacca, 1998). Thus, induction of apoptosis by stress factors including IR might be mediated by different though co-operating signal transduction processes.

We observed a transient G1 arrest in the p53-deficient tumour cells after sphingomyelin pathway activation. Similarly, a G1 arrest of the cell cycle induced by ceramide has been observed in the B lymphoma Raji cells, Hs-27 cells, and Molt-4 cells. Mechanistically, ceramide-induced cell cycle arrest has been linked to an underphosphorylated state of the retinoblastoma protein, pRb, thereby preventing further progression through the S phase upon sequestration of the transcription factor E2F (Jayadev et al, 1995; Kuroki et al, 1996; Alesse et al, 1998). It might be interesting to exploit an even stronger induction of a p53-independent G1 arrest via dose escalation studies with ceramide or drugs interacting with the ceramide pathway in combination with other cytotoxic agents. Specific inhibitors against protein kinase C, which interferes with the sphingomyelin pathway, might be interesting candidates for such investigations (Chmura et al, 1997; Tsuchida and Urano, 1997).

Differences in the apoptotic response after γ-irradiation in the acid sphingomyelinase knockout mice compared to the response in p53 knockout animals suggested that p53- and ceramide-mediated apoptosis are likely distinct and independent (Santana et al, 1996). Furthermore, ceramide-induced apoptosis has been demonstrated in p53-deficient tumour cells, that rapidly undergo apoptosis as stress response to different treatments (e.g. HL-60 cells) (Jarvis et al, 1994). However, the role of sphingomyelinase and p53 for stress-induced apoptosis can vary among various tumour cells and even more so can be different from their untransformed origins (Obeid et al, 1993; Santana et al, 1996). p53-dependent apoptosis in response to ionizing radiation occurs to a large extent only in tumour cells and in cells prone to undergo apoptosis like primary thymocytes (Clarke et al, 1993; Lowe et al, 1993*a*). Our defined cell system is derived from mouse embryo fibroblasts, that in an untransformed status, regardless of the p53 status, does not undergo apoptosis upon irradiation but acquires its strict p53-dependent apoptotic response to chemo- and radiotherapeutical treatment upon oncogenical transformation (Lowe et al, 1993*a*, 1994*b*). Thus, the different p53-dependent biological response to the second messenger ceramide as presented in this study is similar to the difference in chemo- and radiosensitivity observed in multiple human tumours with wild-type and mutated p53 status in vitro and in vivo.

The role of p53 in apoptosis induction has been mainly studied upon DNA-damaging chemotherapeutical drugs and irradiation. However, IR also activates specific signal transduction cascades generated at the plasma membrane. Our results suggest that p53 is not only important in the cellular response to DNA damage, but also for the cellular response to specific apoptotic signal transduction cascades like the sphingomyelin pathway, generated at the plasma membrane.

### ACKNOWLEDGEMENTS

This work was supported by Grants from the Swiss Cancer League, the Zurich Cancer League and the Radiumstiftung (to SB) and the Sassella Foundation (to Y-QS).

### REFERENCES

Alesse E, Zazzeroni F, Angelucci A, Giannini G, Di Marcotullio L and Gulino A (1998) The growth arrest and downregulation of c-myc transcription induced by ceramide are related events dependent on p21 induction, Rb underphosphorylation and E2F sequestering. *Cell Death Diff* **5**: 381–389

Amarante-Mendes GP, Naekyung Kim C, Liu L, Huang Y, Perkins CL, Green DR and Bhalla K (1998) Bcr-abl exerts its antiapoptotic effect against diverse apoptotic stimuli through blockage of mitochondrial release of cytochrome C and activation of caspase-3. *Blood* **91**: 1700–1705

Chinnaiyan AM, Orth K, O'Rourke K, Duan H, Poirier GG and Dixit VM (1996) Molecular ordering of the cell death pathway: Bcl-2 and Bcl-xL function upstream of the CED-3 like apoptotic proteases. *J Biol Chem* **271**: 4573–4576

Chmura SJ, Nodzenski E, Beckett MA, Kufe DW, Quintans J and Weichselbaum RR (1997) Loss of ceramide production confers resistance to radiation-induced apoptosis. *Cancer Res* **57**: 1270–1275

Clarke AR, Purdie CA, Harrison DJ, Morris RG, Bird CC, Hooper ML and Wyllie AH (1993) Thymocyte apoptosis induced by p53-dependent and independent pathways. *Nature* **362**: 849–852

Darzynkiewic Z (1995) In: *Cell Growth and Apoptosis*, Sudzinski GP. Oxford University Press: Oxford

Datta SR, Dudek H, Tao X, Masters S, Fu H, Gotoh Y and Greenberg ME (1997) Akt phosphorylation of BAD couples survival signals to the cell-intrinsic death machinery. *Cell* **91**: 231–241

Fisher DE (1994) Apoptosis in cancer therapy: crossing the threshold. *Cell* **78**: 539–542

Haimovitz-Friedman A, Kan CC, Ehleiter D, Persaud RS, McLouglin M, Fuks Z and Kolesnick RN (1994) Ionizing radiation acts on cellular membranes to generate ceramide and induce apoptosis. *J Exp Med* **180**: 525–535

Haimovitz-Friedman A, Kolesnick RN and Fuks Z (1997) Ceramide signaling in apoptosis. *Br Med Bull* **53**: 539–553

Hannun YA and Linardic CM (1993) Sphingolipid breakdown products: anti-proliferative and tumor-suppressor lipids. *Biochim Biophys Acta* **1154**: 223–236

Hannun YH (1997) Apoptosis and the dilemma of cancer chemotherapy. *Blood* **89**: 1845–1853

Harris CC (1996) Structure and function of the p53 tumor suppressor gene: clues for rational cancer therapeutic strategies. *J Natl Cancer Inst* **88**: 1442–1455

Herr I, Wilhelm D, Böhler T, Angel P and Debatin KM (1997) Activation of CD95 (Apo-1/Fas) signaling by ceramide mediates cancer therapy-induced apoptosis. *EMBO J* **16**: 6200–6208

Jacks T, Remington L, Williams B, Schmitt E, Halachmi S, Bronson R and Weinberg R (1994) Tumor spectrum analysis in p53-mutant mice. *Curr Biol* **4**: 1–7

Jarvis WD, Kolesnick RN, Fornari FA, Traylor RS, Gewirtz DA and Grant S (1994) Induction of apoptotic DNA damage and cell death by activation of the sphingomyelin pathway. *Proc Natl Acad Sci USA* **91**: 73–77

Jarvis WD, Grant S and Kolesnick RN (1996) Ceramide and the induction of apoptosis. *Clin Cancer Res* **2**: 1–6

Jayadev S, Liu B, Bielawska AE, Lee JY, Nazaire F, Pushkareva MY, Obeid LM and Hannun YA (1995) Role for ceramide in cell cycle arrest. *J Biol Chem* **270**: 2047–2052

Kinscherf R, Claus R, Wagner M, Gehrke C, Kamencic H, Hou D, Nauen O, Schmiedt W, Kovacs G, Pill J, Metz J and Deigner HP (1998) Apoptosis caused by oxidized LDL is manganese superoxide dismutase and p53 dependent. *FASEB J* **12**: 461–467

Kuroki J, Hirokawa M, Kitabayashi A, Lee M, Horiuchi T, Kawabata Y and Miura AB (1996) Cell permeable ceramide inhibits the growth of B lymphoma Raji cells lacking TNF-alpha-receptors by inducing G0/G1 arrest but not apoptosis:

© Cancer Research Campaign 1999 

a new model for dissecting cell-cycle arrest and apoptosis. *Leukemia* **10**: 1950–1958
Lowe SW, Schmitt EM, Smith SW, Osborne BA and Jacks T (1993*a*) p53 is required for radiation-induced apoptosis in mouse thymocytes. *Nature* **362**: 847–849
Lowe SW, Ruley HE, Jacks T and Housman DE (1993*b*) p53-dependent apoptosis modulates the cytotoxicity of anticancer agents. *Cell* **74**: 957–967
Lowe SW, Bodis S, McClatchey A, Remington L, Ruley HE, Fisher DE, Housman DE and Jacks T (1994*a*) p53 can determine the efficacy of cancer therapy in vivo. *Science* **266**: 807–810
Lowe SW, Jacks T, Housman DE and Ruley HE (1994*b*) Abrogation of oncogene-associated apoptosis allows transformation of p53-deficient cells. *Proc Natl Acad Sci USA* **91**: 2026–2030
Maity A, Kao GD, Mische RJ and McKenna WG (1997) Potential molecular targets for manipulating the radiation response. *Int J Radiat Oncol Biol Phys* **37**: 639–653
Miyashita T and Reed JC (1995) Tumor suppressor p53 is a direct transcriptional activator of the human bax gene. *Cell* **80**: 293–299
Obeid LN, Linardic CM, Karolak LA and Hannun YA (1993) Programmed cell death induced by ceramide. *Science* **259**: 1769–1771
Ruley HE (1996) In: *Important Advances in Oncology*, DeVita VT, Hellmann S and Rosenberg SA (eds). Lippincott: Philadelphia
Santana P, Pena LA, Haimovitz-Friedman A, McLoughlin M, Cordon-Carlo C, Schuchman EH, Fuks Z and Kolesnick R (1996) Acid sphingomyelinase-deficient human lymphoblasts and mice are defective in radiation-induced apoptosis. *Cell* **86**: 189–199
Suzumiel I (1994) Review: Ionizing radiation-induced cell death. *Int J Radiat Oncol Biol Phys* **66**: 329–341
Tsuchida E and Urano M (1997) The effect of UCN-01 (7-hydroxystaurosporine), a potent inhibitor of protein kinase C, on fractionated radiotherapy or daily chemotherapy of a murine fibrosarcoma. *Int J Radiat Oncol Biol Phys* **39**: 1153–1161
Verheij M, Bose R, Lin XH, Yao B, Jarvis WD, Grant S, Birrer MJ, Szabo E, Zon I, Kyriakis JM, Haimovitz-Friedman A, Fuks Z and Kolesnick RN (1996) Requirement for ceramide-initiated SAPK/JNK signalling in stress-induced apoptosis. *Nature* **380**: 75–79
Wyllie AH (1993) Apoptosis (The Frank Rose Memorial Lecture). *Br J Cancer* **67**: 205–208
Zhou H, Summers SA, Birnbaum MJ and Pittman RN (1998) Inhibition of Akt kinase by cell-permeable ceramide and its implications for ceramide-induced apoptosis. *J Biol Chem* **27**: 16568–16575
Zundel W and Giaccia A (1998) Inhibition of the anti-apoptotic PI(3)K/Akt/Bad pathway by stress. *Genes Dev* **12**: 1941–1946

 *© Cancer Research Campaign 1999*
